# Rotavirus-Associated Myocarditis in an Immunocompetent Adult: A Case Report

**DOI:** 10.7759/cureus.75093

**Published:** 2024-12-04

**Authors:** Jungwuk Han

**Affiliations:** 1 Internal Medicine, Weiss Memorial Hospital, Chicago, USA

**Keywords:** acute systolic heart failure, case report, perimyocarditis, rotavirus, viral-induced myocarditis, viral pericarditis

## Abstract

A previously healthy, 28-year-old man presented with a two-day history of diarrhea and chest pain, suggestive of infectious myocarditis. Initial workup revealed elevated troponin-I levels and diffuse ST-segment elevations on electrocardiogram (ECG). Transthoracic echocardiography showed a reduced left ventricular ejection fraction (40-45%), posteroinferior wall akinesis, and a small pericardial effusion. Stool studies were positive for rotavirus antigen via enzyme immunoassay (EIA). Cardiac magnetic resonance imaging (MRI) and endomyocardial biopsy (EMB) were not performed due to facility limitations; however, clinical findings, troponin-I trends, and echocardiographic abnormalities supported the diagnosis of rotavirus-associated myocarditis. The patient was treated with oral rehydration, colchicine, and metoprolol, leading to symptom resolution and a decline in troponin-I levels. This case underscores the importance of considering rotavirus as a potential etiologic agent in myocarditis, even in immunocompetent adults without significant comorbidities, and highlights the need for clinicians to recognize gastrointestinal viruses as possible causes of cardiac inflammation.

## Introduction

Myocarditis, characterized by myocardial inflammation, may arise from both infectious and noninfectious etiologies. Viral infections are among the most common causes, especially parvovirus B19 and human herpesvirus 6. Nonviral origins include infections such as Lyme disease and Chagas disease, in addition to certain medications known to provoke hypersensitivity eosinophilic myocarditis [1].

Rotavirus, a double-stranded, non-enveloped RNA virus predominantly affecting the gastrointestinal system, is chiefly recognized as a cause of gastroenteritis in children and is rarely implicated in adult myocarditis. Recent studies indicate that rotavirus infection may contribute to a spectrum of systemic conditions, including neurological disorders, hepatitis, type 1 diabetes, respiratory illnesses, myocarditis, and renal dysfunction, with molecular research elucidating potential pathogenetic mechanisms [2]. Rotavirus may cause myocarditis through direct viral invasion of myocardial tissue or immune-mediated mechanisms involving cytokine release and systemic inflammation. The systemic inflammatory response triggered by rotavirus gastroenteritis, along with elevated pro-inflammatory cytokines, may contribute to myocardial inflammation and damage.

Cases of rotavirus-associated myocarditis in adults are exceedingly rare, with only two histologically confirmed cases reported in the literature, both involving patients with preexisting cardiac conditions [3]. This underscores the significance of recognizing rotavirus as a potential etiologic agent in myocarditis, even in immunocompetent adults without significant comorbidities.

This case report seeks to expand the limited body of literature on rotavirus-associated myocarditis, underscoring the importance of accurate diagnosis and effective management, particularly given the potential for severe complications in patients with preexisting cardiac conditions and the need for clinicians to consider gastrointestinal viruses as possible causes of cardiac inflammation.

## Case presentation

**Figure 1:**
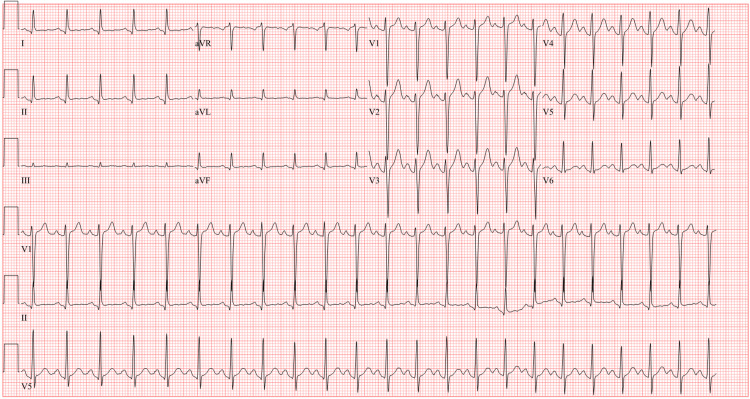
Initial ECG upon presentation to the emergency department, demonstrating sinus tachycardia without significant ST-segment changes

A 28-year-old male with a past medical history of pericarditis following a diarrheal illness in childhood and intermittent asthma presented to the emergency department (ED) with two days of nausea and diarrhea, along with one day of chest pain. Initial assessment included a complete blood count (CBC), comprehensive metabolic panel (CMP), electrocardiogram (ECG), and troponin-I. The ECG demonstrated sinus tachycardia with a heart rate of 136 bpm, without ST-segment changes (Figure 1). The troponin-I level was 0.01 ng/mL, which was within the normal range. The patient's symptoms were managed with ketorolac, ondansetron, and intravenous fluids, and he was discharged with ondansetron as needed, given the absence of significant cardiac findings.

Within 24 hours, the patient experienced recurrent chest pain unrelieved by ibuprofen, prompting a return to the ED. He reported approximately 10 episodes of watery, non-bloody diarrhea daily. His chest pain, which began one day after the onset of diarrhea, was intermittent, pressure-like, radiated to the upper back, and worsened with deep inspiration. He did not report any positional changes in chest pain or relief with leaning forward.

The patient denied vomiting, fever, chills, dyspnea, cough, sputum production, rhinorrhea, abdominal pain, peripheral edema, joint pain, and rash. He worked in an overcrowded shelter with suboptimal hygiene conditions, where he was recently exposed to others with diarrheal illness. He denied tobacco use, excessive alcohol consumption, and illicit drug use and reported no known allergies.

During this visit, the physical examination was notable only for sinus tachycardia with a heart rate of 120 bpm and mild dehydration. Cardiac auscultation revealed regular S1 and S2 heart sounds, with no friction rubs or murmurs. Pulmonary examination demonstrated clear breath sounds bilaterally. The abdomen was non-tender with normoactive bowel sounds, and there was no evidence of tenderness on palpation of the chest or abdomen. Extremity examination showed no signs of edema.

**Figure 2:**
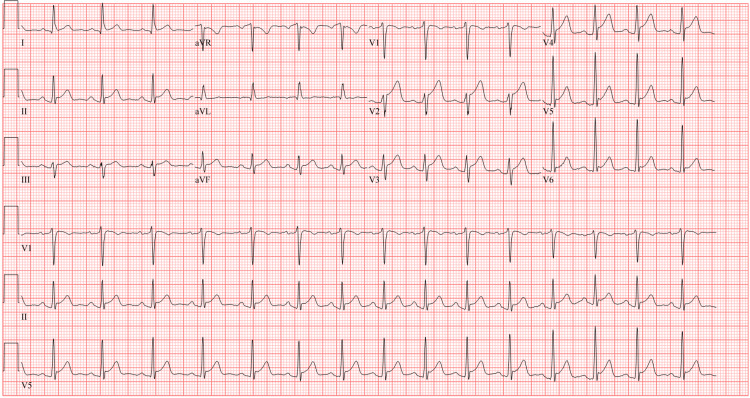
Follow-up ECG obtained 24 hours post-presentation, showing sinus tachycardia with diffuse ST-segment elevations in leads V2-V6, II, III, and aVF

Repeat workup revealed significant ECG changes, with diffuse ST elevations in leads V2-V6, II, III, and aVF (Figure 2), correlating with myopericarditis rather than acute coronary syndrome. Chest X-ray showed no acute pathology, specifically ruling out cardiomegaly and pulmonary edema. Blood tests revealed a markedly elevated troponin-I of 2.33 ng/mL, up from 0.01 ng/mL recorded 22 hours prior. Other cardiac markers, such as creatine kinase-MB (CK-MB) and brain natriuretic peptide (BNP), were not evaluated at that time. Based on the clinical history and findings, a diagnosis of myopericarditis was established. Treatment with colchicine, ibuprofen, and metoprolol was initiated, and the patient was admitted to the hospital.

**Figure 3:**
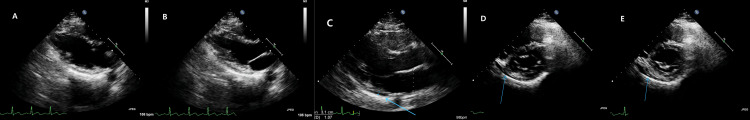
Transthoracic echocardiographic views demonstrating postero-inferior wall akinesis, moderate global hypokinesis, a reduced left ventricular ejection fraction (estimated at 40–45%), and a small pericardial effusion (indicated by the blue arrow).

The patient continued to experience diarrhea, with a dramatic rise in troponin-I to 36.26 ng/mL after 12 hours. ECG findings remained consistent, showing diffuse abnormalities without specific localization. Transthoracic echocardiography (TTE) revealed postero-inferior wall akinesis, a reduced left ventricular ejection fraction of 40-45%, moderate diffuse hypokinesis, and a small pericardial effusion (Figure 3). No prior echocardiograms were available for comparison to determine if these findings were new or residual.

Cardiology was consulted to perform a coronary angiogram to exclude coronary vascular causes of myocardial injury. Left heart catheterization with coronary angiography revealed no evidence of epicardial coronary artery disease and normal left ventricular filling pressures. The elevated troponin-I levels and myocardial injury were thus attributed to myocarditis secondary to an infectious etiology. Although cardiac MRI or endomyocardial biopsy is considered the gold standard for confirming myocarditis, these tests were not performed due to facility limitations and rapid clinical improvement. The diagnosis relied on clinical findings, troponin-I trends, and echocardiographic abnormalities consistent with myocardial injury.

Following the diagnosis of myocarditis and heart failure with reduced ejection fraction, treatment was continued only with colchicine and metoprolol. ACE inhibitors were not initiated during the acute phase, anticipating rapid resolution of heart dysfunction and the absence of other compelling indications. Diuretics were not administered due to the absence of significant fluid overload and related symptoms. Colchicine was chosen for its anti-inflammatory effects in pericarditis, as it disrupts microtubule polymerization, reducing inflammatory cell migration and cytokine release [4]. Metoprolol was used to manage heart failure symptoms and provide rate control.

Subsequent troponin-I levels declined to 28.13 ng/mL and later to 8.62 ng/mL. The patient's diarrhea spontaneously resolved the following day, with no recurrence of chest pain. Stool studies confirmed a positive rotavirus antigen via enzyme immunoassay (EIA). The EIA test for rotavirus antigen detection has high specificity (100%) and sensitivity ranging from 75% to 82.1%, reinforcing the diagnostic reliability [5]. Other pathogens or etiologies for diarrhea, such as bacterial or parasitic infections, were considered less likely given the absence of fever, abdominal pain, or bloody stools, and were not detected in stool studies.

He had an uneventful hospital course and was discharged with contact precautions, including recommendations for environmental cleaning and hygiene measures to prevent transmission [6]. Follow-up was recommended with his primary care provider and cardiologist, including repeat echocardiograms at one and three months to assess ejection fraction recovery and monitor for potential arrhythmias or other sequelae.

## Discussion

Distinguishing myocarditis from pericarditis is critical, though the conditions are not mutually exclusive. The terms "perimyocarditis" or "myopericarditis" are often used to denote the concurrent presence of both conditions, depending on the primary pathologic process. The overlap between myocarditis and pericarditis complicates diagnosis as both conditions share common symptoms (e.g., chest pain, ECG abnormalities) and may coexist.

In this case, the patient presented with concurrent findings of myocarditis and pericarditis, evidenced by diffuse ST-segment elevations, elevated troponin levels, and pericardial effusion on echocardiography. The temporal progression of symptoms, with chest pain following gastrointestinal illness, supported an infectious etiology.

Rotavirus-related myocarditis in adults is exceedingly rare, as aforementioned [3]. Although somewhat more common in young children, cases remain infrequent, and clinical outcomes can vary [7,8]. Mild elevations in CK-MB levels have been documented in pediatric cases, potentially indicating myocardial inflammation [9].

Rotavirus may cause myocarditis through direct viral invasion of myocardial tissue or immune-mediated mechanisms involving cytokine release and systemic inflammation. The systemic inflammatory response triggered by rotavirus gastroenteritis, along with elevated pro-inflammatory cytokines, may contribute to myocardial inflammation and damage [2,10].

When myocarditis is suspected, a diagnostic evaluation should aim to exclude more prevalent conditions, such as atherosclerotic and valvular heart diseases. The initial diagnostic approach typically involves serum biomarkers, including troponin. Non-specific indicators, such as elevated C-reactive protein and ECG abnormalities (e.g., sinus tachycardia, ST-segment abnormalities), may support a diagnosis of myocarditis but are of limited specificity. Echocardiography assists in excluding other cardiac pathologies while cardiac MRI offers a sensitive and non-invasive confirmation method. The Lake Louise criteria for cardiac MRI provide diagnostic sensitivity for myocarditis. However, cardiac MRI and endomyocardial biopsy (EMB), which remains the diagnostic gold standard, were not performed in this case due to facility limitations and rapid clinical improvement. EMB is typically reserved for severe or refractory cases or when other methods are inconclusive, as it provides histological, immunohistochemical, and molecular insights crucial for understanding the underlying etiology and guiding targeted therapy [10,11].

This patient had a history of pericarditis following a diarrheal illness in childhood, leaving uncertain the extent of any pre-existing myocardial damage prior to the current episode. The lack of prior echocardiograms precludes definitive assessment of baseline cardiac function. However, the marked elevation in troponin-I levels, notable ECG changes, and findings from TTE and coronary angiography strongly indicate an acute myocardial injury secondary to rotavirus infection.

Management of myocarditis is guided by clinical presentation and associated complications, such as heart failure and arrhythmias, which are treated supportively in accordance with established guidelines. Routine use of immunosuppressive therapy is not recommended unless biopsy-proven autoimmune myocarditis is diagnosed. Colchicine, administered for up to three months, may alleviate chest pain related to concomitant pericarditis. It exerts its therapeutic effects by disrupting microtubule polymerization, reducing inflammatory cell migration and cytokine release [4]. Non-steroidal anti-inflammatory drugs (NSAIDs) should be used cautiously, typically reserved for patients with preserved ventricular function [11].

Rotavirus is rarely implicated as a cause of myocarditis. This case highlights the rarity of rotavirus-associated myocarditis in immunocompetent adults without significant cardiac comorbidities. It underscores the importance of recognizing gastrointestinal viruses as potential causes of cardiac inflammation, even in patients without significant comorbidities. Clinicians should maintain a high index of suspicion for myocarditis in patients presenting with viral symptoms and chest pain, and consider appropriate diagnostic evaluations and management strategies.

## Conclusions

This case details a 28-year-old immunocompetent male without significant cardiac comorbidities who was diagnosed with rotavirus-associated myocarditis. Despite the absence of cardiac MRI and endomyocardial biopsy, the diagnosis was supported by clinical findings, troponin-I trends, and echocardiographic abnormalities. The patient's condition improved under medical management with oral rehydration, colchicine, and metoprolol, leading to symptom resolution and a decline in troponin-I levels. He was discharged with follow-up recommendations and contact precautions. This case underscores the importance of considering rotavirus as a potential etiologic agent in myocarditis, even in immunocompetent adults. Clinicians should recognize the potential systemic impact of rotavirus and maintain a high index of suspicion for myocarditis in patients presenting with gastrointestinal symptoms and chest pain. The combination of oral rehydration, colchicine, and beta-blockers aligns with current myocarditis management guidelines and was effective in resolving the patient's symptoms.
